# Lung microbiota in COPD: a double-edged sword

**DOI:** 10.1016/j.crmicr.2026.100679

**Published:** 2026-10-30

**Authors:** Abderrazzak Bentaher

**Affiliations:** Equipe « Inflammation et Immunité de l’Epithélium Respiratoire », INSERM, UR-3738 « CICLY», Faculté de Médecine Lyon Sud, UCBL1, France

**Keywords:** Microbiota, Lung, COPD, Dysbiosis

## Abstract

The respiratory microbiota constitutes a dynamic, low-biomass microbial ecosystem whose composition and function are shaped by continuous immigration, elimination, local growth conditions and host-environment interactions. Chronic obstructive pulmonary disease (COPD) provides a clinically important model in which this ecological equilibrium is profoundly altered. This review examines the lung microbiota through a “double-edged sword” framework, proposing that its biological effects depend less on the presence of individual beneficial or pathogenic taxa than on community structure, ecological stability, microbial function and host context. We examine the organization of the lung microbial ecosystem in health and its transition toward dysbiotic, unstable and pathobiont-enriched states in COPD, together with the host, environmental and therapeutic factors that shape these transitions. Particular attention is given to microbial community dynamics, functional alterations, epithelial and immune interactions, antimicrobial defense, and the contribution of the gut-lung microbial axis. We further address the unresolved directionality of the dysbiosis-COPD relationship, considering evidence that COPD-associated changes in the airway environment can select for altered microbial communities while established dysbiosis may subsequently reinforce disease-associated conditions, generating a self-perpetuating ecological cycle. Longitudinal profiling, metagenomics, metatranscriptomics, metabolomics and integrated host-microbiome approaches are discussed as means of moving beyond taxonomic associations toward microbial function and mechanism. Finally, we critically evaluate emerging microbial signatures with potential prognostic or treatment-response relevance and microbiota-targeted interventions, while emphasizing current limitations in causality, methodological standardization, strain-level resolution and clinical validation. A microbiota-centered perspective may therefore help clarify how changes in the structure and function of the lung microbiota contribute to the transition from pulmonary homeostasis to COPD-associated dysbiosis and disease progression.

## Highlights

• Lung microbiota can promote both tissue homeostasis and disease-associated responses.
• COPD dysbiosis reflects shifts in microbial structure, function and stability.
• Host, environmental and treatment factors shape the lung microbial ecosystem.
• Lung dysbiosis may reinforce epithelial dysfunction and immune dysregulation.
• Longitudinal and multi-omics studies are key to defining microbiota causality.

## Graphical abstract

Figure. The double-edged role of the lung microbiota in COPD. A balanced lung microbiota ecosystem supports homeostasis through barrier integrity, immune equilibrium, and controlled inflammation. Dysbiosis-driven by host, environmental and therapeutic factors disrupts these functions and promotes inflammation, tissue injury, exacerbations and disease progression. COPD and dysbiosis engage in bidirectional, self-reinforcing cycle. Host exposures, infections, therapies, and the gut-lung axis are shown as ecological modifiers.

**Figure:**
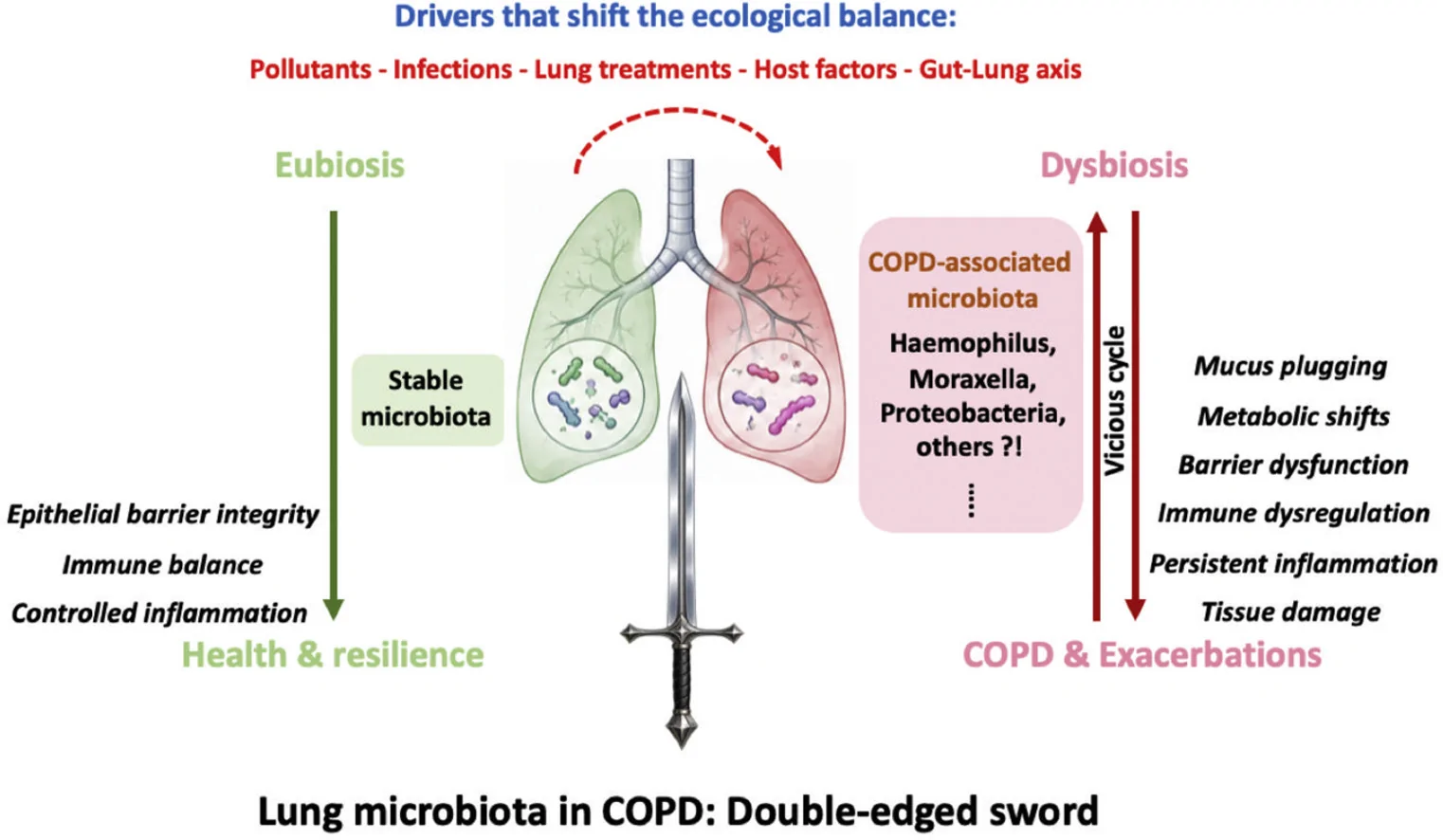

## Scope and terminology

The terms “microbiota” and “microbiome” are related but not synonymous. Microbiota refers to the microorganisms present in a defined habitat, whereas microbiome encompasses their collective genetic material and, more broadly, their functional potential. This distinction is particularly relevant in respiratory research because taxonomic composition alone does not capture microbial activity or host-microbe interactions (Marchesi and Ravel, 2015).

### Development of lung microbiota research

Microbiota research has evolved from culture-based descriptions of host-associated microorganisms to culture-independent profiling enabled by 16S rRNA gene sequencing, metagenomics, and related omics technologies. These approaches established that microbial communities can coexist with the host and have enabled large-scale initiatives such as the Human Microbiome Project, providing the methodological foundation for contemporary investigation of low-biomass ecosystems such as the lung (Berg et al., 2020).

### Lung microbiota

Recent advances in sequencing technologies have revealed a diverse and dynamic microbial community, collectively referred to as the lung microbiota. This community is composed of bacteria, viruses, and fungi that colonize the respiratory tract, influenced by factors such as age, environment, and host immunity. While the composition of the lung microbiota could differ from that of the gut, it plays a critical role in pulmonary homeostasis and disease pathogenesis such as chronic obstructive pulmonary disease (COPD) (this review) (Li et al., 2024, Zhou et al., 2024).

### Microbiota roles in healthy lungs

Rather than being sterile as previously thought, it has been largely documented that healthy lungs contain diverse microbial populations spreading out from the upper airways to the alveoli and exhibiting defined functions (Dickson et al., 2016). It has been also revealed that lung microbiota, while in a relatively low biomass, includes bacteria (e.g., Prevotella, Veillonella, Streptococcus, Neisseria, Haemophilus, *Pseudomonas*), fungi (mycobiota) (e.g., Candida, Aspergillus), viruses (virome) (e.g., bacteriophages and resident viruses) and in traces, archaea and protozoa as well (Charlson et al., 2011; Man et al., 2017). These microorganisms live usually in a symbiotic relationship (e.g. mutualistic, commensal) with lung constituents. Importantly, the biological effect of a given taxon is context-dependent: organisms regarded as commensal in one ecological setting may participate in inflammation or disease when community structure or host conditions change. This ecological plasticity underpins the “double-edged sword” framework developed throughout this review. Below are different biological roles that have been attributed to lung microbiota, which contribute to the maintenance of healthy lungs (Fig. 1).

**Fig. 1:**
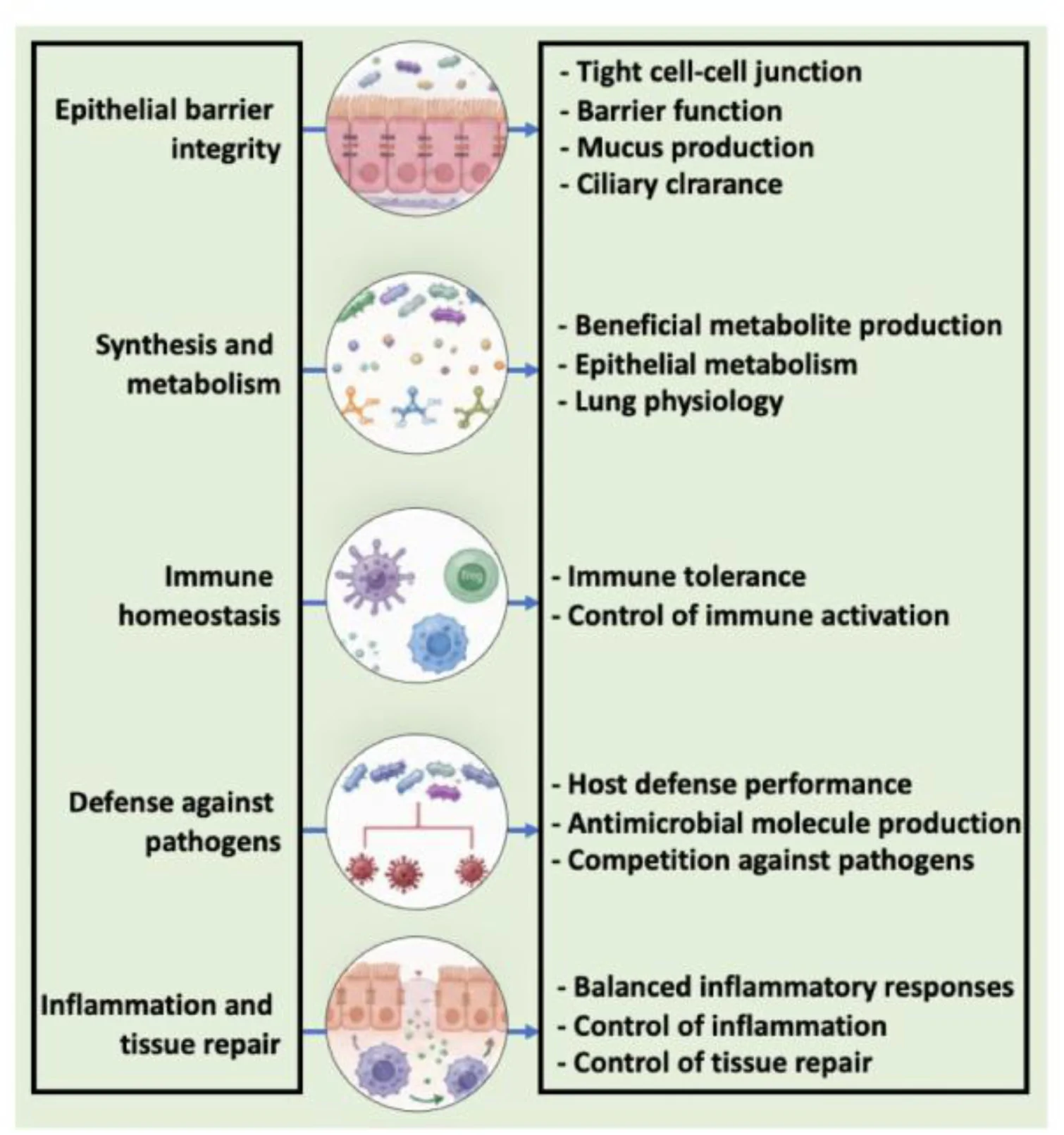
Role of the lung microbiota in pulmonary homeostasis. Left panel: in healthy lungs, a balanced microbiota may contribute to epithelial barrier integrity, microbial metabolic activity, immune homeostasis, colonization resistance, antimicrobial defense, and regulation of inflammation and tissue repair. Right panel: microbiota could shape various lung physiologic functions*.*

### Epithelial barrier integrity

Emerging evidence suggests that respiratory microbial communities interact with epithelial pathways involved in mucus production, junctional integrity, epithelial renewal, and mucociliary function. However, direct evidence supporting some of these functions in the human lung remains limited compared with the gut, emphasizing that they arise from dynamic interactions between the respiratory microbiota and the host rather than from microbial activity alone (Man et al., 2017; Dickson et al., 2014; Budden et al., 2017).

### Synthesis and metabolism

The primary site for these functions is the gut, but the capacity of lung microbiota to synthesize or metabolize substrates in situ is less well characterized. Respiratory bacteria can harbor genes involved in vitamin and other metabolic pathways, including pathways associated with Streptococcus, Prevotella, and Veillonella (Dickson et al., 2016). Streptococcus, Prevotella, and Veillonella harbor metabolic potential to synthesize vitamin B group (e.g., B2, B6, folate) that could regulate immune cell function and epithelial integrity (Kjer-Nielsen et al., 2012). For example, metabolites of vitamin B2 (Riboflavin) can activate mucosal responses. Veillonella species could contribute to metabolic signaling, though specific vitamin production in the lung remains under investigation (Kjer-Nielsen et al., 2012). Other bioactive compounds that are produced directly or indirectly by the lung microbiota include short-chain fatty acids (SCFAs) (e.g., acetate, propionate, butyrate) whose functions are proposed to involve anti-inflammatory role and modulation of alveolar macrophage properties. Much of the mechanistic evidence derives from the gut-lung axis rather than local pulmonary production (Dang and Marsland, 2019). With respect to lipid metabolism, *microbiota-derived phospholipids* may influence host-surfactant-related pathways and lipid homeostasis, although this remains less well defined. Indirectly, lung microbiota might induce host antimicrobial peptides involved in defense and microbial balance (Dumas et al., 2018). Interestingly, alterations in bioactive compounds-producing lung microbiota have been associated with diseases such as chronic obstructive pulmonary disease (COPD) (see below). Thus, functional activity, rather than taxonomic presence alone, represents an important but still incompletely defined dimension of lung microbial ecology.

### Immune homeostasis and modulation

A dynamic relationship between lung microbiota and immune system is at play to ensure both a normal respiratory health and/or defense against disease development. As mentioned above, it is indeed documented that healthy lungs host a diverse microbial community at low biomass (Whiteside et al., 2021)*.* The nonstop microbial exposure contributes to immune education helping the immune system to discern between harmless and harmful organisms by shaping innate and adaptive immune responses and promoting immune tolerance (Dickson et al., 2016; Belkaid and Hand, 2014). Hence, this lung microbiota-mediated balance of immune activation and tolerance supports the ability of the lungs to respond to infection while limiting excessive inflammatory responses to insulting agents like dust. Mechanistically, this implies that microbial components like lipopolysaccharides or peptidoglycans stimulate pattern-recognition receptors (PRRs) such as toll-like receptors (TLRs) and nucleotide-binding oligomerization domain (NODs) on immune cells to promote immune system regulation (Dickson et al., 2016; Huffnagle et al., 2017; Takeuchi and Akira, 2010). Not to mention that when pathogens are detected, the microbiota “primes” the innate immune system to respond faster.

### Protection against pathogens

The lung microbiota contributes to protection against invading pathogens through ecological and immune-mediated mechanisms. Through colonization resistance, resident microbial communities occupy ecological niches and compete with potential pathogens for available nutrients and attachment sites, thereby limiting microbial invasion and maintaining ecosystem stability (Man et al., 2017; Dickson et al., 2016). Respiratory commensals may also produce antimicrobial molecules and metabolites that influence pathogen growth; however, the extent to which bacteriocin production by lung microbiota contributes to respiratory protection remains incompletely defined (Man et al., 2017). In addition, the lung microbiota contributes to host defense by shaping innate and adaptive immune responses through interactions with epithelial cells, macrophages, dendritic cells, and other immune populations, thereby promoting effective responses to bacterial, viral, and fungal infections (Budden et al., 2017; Dickson et al., 2016).

### Regulation of inflammation and tissue repair

Rather than acting as an isolated regulator, the lung microbiota participates in a reciprocal ecological system in which microbial immigration, mucociliary clearance, environmental exposure, host immunity, inflammation, and tissue repair continuously interact (Dickson et al., 2016; Man et al., 2017; Huffnagle et al., 2017). Under homeostatic conditions, these interactions may support controlled inflammatory responses and recovery after injury; when the airway environment changes, the same ecological relationships may instead favor persistent inflammation and dysbiosis.

### Lung-Gut crosstalk

There is a bidirectional relationship between the lungs and the gut involving their respective microbiota (Fig. 2) (Marsland et al., 2015; Yu et al., 2025; Sun et al., 2024; Trompette et al., 2014). As such, there is a reciprocal impact on each other’s health and function. The beneficial effects include modulation of the immune system responses via circulating inflammatory mediators such as cytokines and chemokines. It has been also reported that lung microbiota may contribute to promote regulatory T cell (Treg) activity, hence maintaining an immune balance in the gut (Budden et al., 2017). Conversely, gut microbiota enhances differentiation of Treg and production of IL-10, reducing thereby excessive airway inflammation and mounting balanced antiviral and antibacterial responses (Trompette et al., 2014). Work by Ichinohe et al. showed that germ-free mice, thus no gut microbiota, exhibited impaired antiviral defenses and severe lung inflammation after influenza infection (Ichinohe et al., 2011). Metabolites (e.g., SCFAs) directly or indirectly produced by both organ microbiota circulate systemically reaching each other epithelia to strengthen their corresponding barriers and reducing permeability. Gut-derived acetate, propionate, and butyrate reduce levels of pro-inflammatory cytokines (e.g., IL-6, TNF-α), improve alveolar macrophage pathogen clearance and lower the susceptibility to airway inflammation. Interestingly, healthy lung and gut microbiota prevent systemic spread of pathogens or inflammatory mediators that could disturb the ecosystem of these organs. Indeed, it has been shown in animal studies that lung colonization with the commensal Lactobacillus spp. increased gut microbial diversity and enhanced resistance to gut infections. In the same vein, gut microbes modulate systemic antimicrobial peptide production while competing with pathogenic organisms. Consequently, this reduces translocation of gut-derived pathogens to the lungs. It also enhances mucosal immunity through for instance production of secretory IgA and defensins, which protect the respiratory system (Dang and Marsland, 2019). It must be emphasized, however, that this bidirectional communication between the intestinal and respiratory systems within the gut-lung axis, if imbalanced, could lead to dysbiosis that predisposes the host to lung and gut diseases such as COPD and colitis, respectively.

**Fig. 2:**
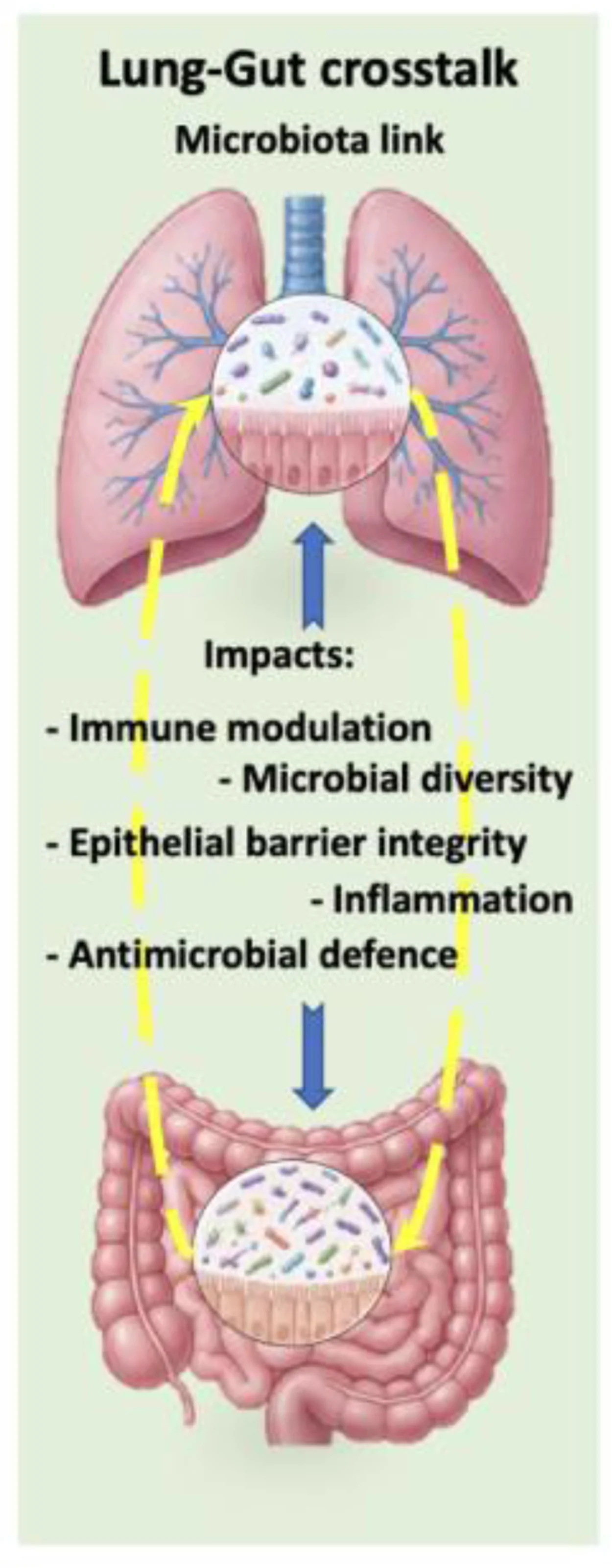
Microbiota-mediated Lung-Gut crosstalk. The intestinal and respiratory ecosystems are connected through a bidirectional communication network (immune and metabolic pathways) in which their respective microbiota impact local and systemic responses.

## Dysbiosis

Dysbiosis refers to an imbalance or a disruption in the composition, diversity, and/or functional capacity of the host-associated microbiota, when compared with a healthy (homeostatic) reference state. This means a shift away from a balanced microbiota (sometimes called *eubiosis*) toward a state where beneficial microbes decline and pathogenic or opportunistic microbes increase, all of which contribute to disease pathogenesis (Tiffany and Bäumler, 2019; Shen et al., 2025). In COPD, this concept should not be reduced to a simple loss of “beneficial” organisms and gain of “pathogens.” Rather, airway dysbiosis reflects altered immigration, elimination, local growth conditions, community interactions, and functional activity, often accompanied by enrichment of potentially pathogenic taxa (Sin, 2023). The consequences are context-dependent and may both result from and reinforce airway inflammation, epithelial injury, and defective clearance. Various intrinsic and extrinsic factors to the host further amplify dysbiosis, creating a self-perpetuating cycle of microbial imbalance and airway inflammation.

### COPD

In this review, we focus on COPD and the role of microbial dysbiosis in disease pathogenesis.

The Global Initiative for Chronic Obstructive Lung Disease (GOLD) provides the most widely adopted framework for defining and stratifying COPD. Thus, GOLD defines COPD as a heterogeneous, preventable, and treatable disease characterized by persistent airflow limitation and chronic respiratory symptoms, typically resulting from airway and/or alveolar abnormalities (26). This chronic respiratory disease is primarily resulting from long-term exposure to noxious particles or gasses, especially cigarette smoke and environmental pollutants. The pathological hallmarks of COPD include chronic airway inflammation, structural airway remodeling, emphysematous destruction of alveolar tissue, oxidative stress, and protease-antiprotease imbalance, driven by interactions between innate and adaptive immune responses and systemic inflammatory processes (Bentaher et al., 2025). Beyond these classical mechanisms, COPD is increasingly recognized as a multisystem condition with significant extrapulmonary influences on disease progression.

Traditionally viewed as a disorder driven by cigarette smoke-induced lung injury and chronic inflammation, COPD pathogenesis also involves intricate host-microbe interactions that influence tissue homeostasis. Indeed, emerging evidence highlights the role of the microbiota, both within the airways and systemically, in modulating immune responses and inflammatory pathways contributing to the complex pathogenesis of COPD (Fig. 3) (Wang et al., 2016; Wypych et al., 2019).

**Fig. 3:**
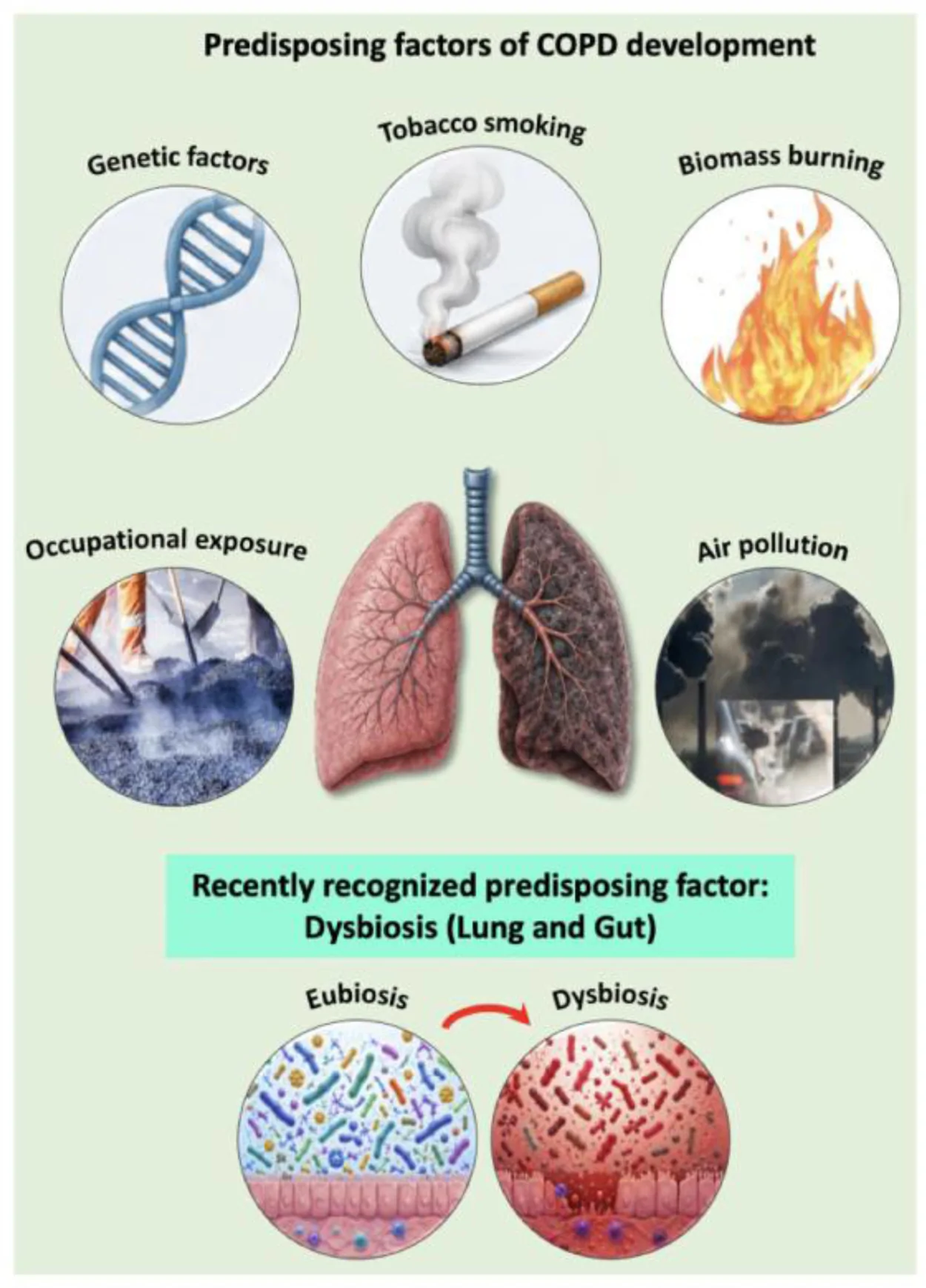
Schematic representation of factors contributing to COPD pathogenesis. Classical risk factors including tobacco smoking, air pollution, occupational and biomass exposures, and genetic predisposition contribute relatively to disease development. In recent years, dysbiosis of lungs and gut has been proposed as novel contributing factor linking microbial imbalance to COPD pathogenesis.

The lung harbors a diverse microbial community even in health, and perturbations in this community have been documented in COPD patients, with shifts toward increased relative abundance of Proteobacteria phylum and decreases in commensal taxa, independent of smoking history, suggesting microbiota alterations are linked with airway inflammation and disease progression rather than merely smoking exposure alone (Haldar et al., 2020). Respiratory dysbiosis in COPD appears to modulate local responses and sustain chronic airway inflammation. Overall, altered lung microbial communities may interact with innate and adaptive immune pathways via pattern recognition receptors and pro-inflammatory signaling, exacerbating tissue injury, mucus hypersecretion, and impaired pathogen clearance; all of which represent characteristic features of COPD pathology (Budden et al., 2017; Wypych et al., 2019). Studies have also shown that dysbiosis is associated with disease severity, frequency of acute exacerbations, and adverse clinical outcomes, underscoring its relevance to key clinical phenotypes of COPD (Huang et al., 2014).

Beyond the lungs, gut microbiota dysbiosis, characterized by reduced diversity and altered metabolic profiles, has emerged as a systemic factor influencing COPD pathogenesis via the gut-lung axis. This bidirectional communication network involves microbial metabolites, immune modulation, and systemic inflammatory signaling pathways that link gut microbial composition with pulmonary immune and inflammatory responses. Preclinical and clinical studies demonstrated that in COPD, the gut exhibits distinct microbial profiles characterized by altered diversity and reduced levels of beneficial metabolites such as short-chain fatty acids. These changes correlate with heightened systemic inflammation and immune dysregulation (Bowerman et al., 2020; Lai et al., 2022). Of relevance, murine studies reported that transplantation of fecal microbiota from COPD patients into recipient mice led to increased lung inflammation, airway remodeling, emphysema, and accelerated declines in lung function, reinforcing the hypothesis of a relationship between gut dysbiosis and COPD-like pathology in vivo (Lai et al., 2022).

Noxious exposures such as cigarette smoke that lead to COPD development likely perturb both lung and gut microbiota (Biedermann et al., 2013). For instance, smoke exposure induces microbial shifts and reduces microbial diversity, which in turn is associated with increased pro-inflammatory cytokine production and oxidative stress, thereby linking environmental risk factors with dysbiosis and disease processes at multiple biological levels . Dysbiosis-mediated modulation of immune cell populations and microbial metabolite signaling likely contributes to the persistent low-grade inflammation that typifies COPD, bridging traditional pathogenic pathways with microbiota-associated mechanisms (Wang et al., 2016; Wypych et al., 2019).

In sum, such dysbiosis is therefore considered as a potential mechanism contributing to disease initiation, progression, and probably exacerbation through local and systemic immune modulation and metabolic dysregulation. Understanding these microbiota-mediated pathways offers promising avenues for novel diagnostic biomarkers and microbe-targeted therapeutic strategies in COPD management (see below).

### From homeostasis to dysbiosis in COPD

In COPD, lung dysbiosis could disrupt pulmonary homeostasis by altering a number of processes (Fig. 4). As such, this results in maladaptive immune and abnormal inflammatory responses and impaired clearance mechanisms including pathogens (Wypych et al., 2019). Dysbiotic microbial communities could also drive sustained airway inflammation (Kayongo et al., 2023). Concurrently, epithelial barrier integrity is compromised facilitating microbial persistence and epithelial injury (Raby et al., 2023) and antimicrobial defenses are weakened by impairing antimicrobial peptide activities and coordinated epithelial-immune responses, thereby increasing susceptibility to infections and acute exacerbations (Ji et al., 2026).

**Fig. 4:**
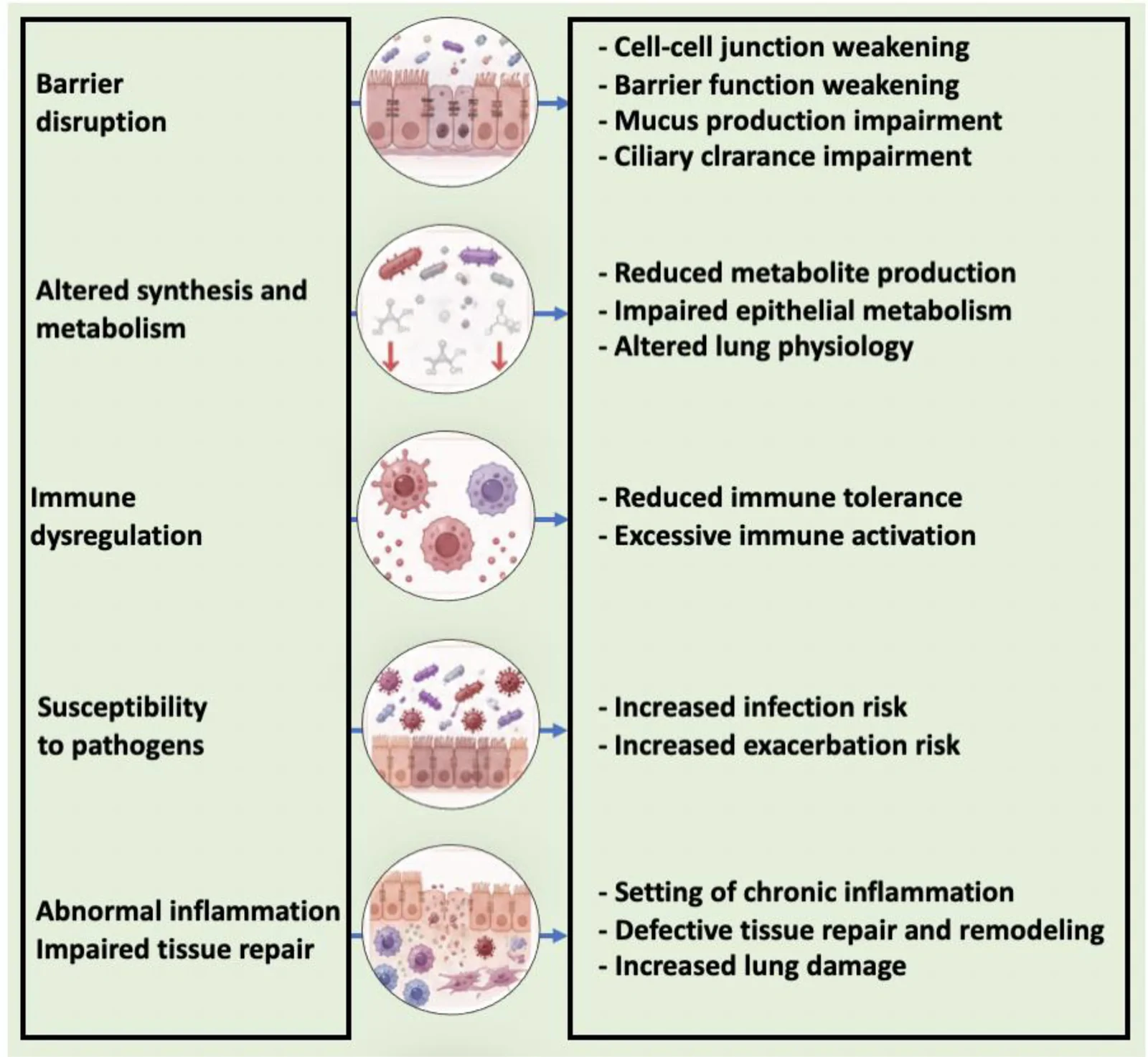
Potential contribution of lung microbiota dysbiosis to COPD pathophysiology. Potential links between lung dysbiosis and impaired airway homeostasis. The left side identifies the main processes that are affected, while the right side summarizes their potential consequences for airway barrier function, microbial metabolism, immune regulation, pathogen susceptibility, inflammation, and tissue repair. These interconnected processes may contribute to persistent airway inflammation and lung damage, potentially reinforcing microbial imbalance. The figure presents a conceptual model, and the extent to which these mechanisms directly contribute to COPD progression in humans remains to be established.

### Ecological basis of the double-edged sword

The “double-edged sword” concept refers to a context-dependent shift in microbial effects rather than to a fixed distinction between beneficial and harmful organisms. The same taxon may have different consequences depending on its abundance, strain, metabolic activity, interactions with neighboring organisms, airway niche, mucus environment, host immune state, and treatment exposure. At the community level, loss of microbial diversity or antagonistic interactions may reduce ecological resilience and permit expansion of disease-associated taxa. At the taxon level, a microorganism commonly regarded as commensal may become pro-inflammatory when host barriers, mucus properties, or immune regulation are altered. This framework generates testable predictions: disease-associated microbial functions should be more reproducible than simple taxonomic labels. Longitudinal shifts in community stability should precede or accompany changes in inflammatory phenotype. And experimentally, restoring specific functions or ecological interactions should improve host phenotypes even when the community does not return to an identical “healthy” composition (Wang et al., 2021; Wang et al., 2020; Yan et al., 2022; Sulaiman et al., 2023).

### Dysbiosis and immune imbalance

Normally, commensal bacteria interact with innate and adaptive immune cells to maintain a balanced immune environment. As mentioned above, dysbiosis involves altered microbial diversity marked by a reduction in beneficial commensals and growth of pathogenic bacteria (e.g. *Haemophilus* and *Moraxella* species). Recent evidence suggests that in COPD, microbiota dysbiosis disrupts the “normal” crosstalk (i.e., MAMP-PPR interactions) between commensal microbes and the immune system in the airways modulating thereby ensuing lung responses.

### Dysbiosis-driven aberrant innate immune activation

The innate immune system represents the first line of defense in the respiratory tract. Under physiological conditions, airway epithelial cells, alveolar macrophages, and dendritic cells interact with commensal microbes to maintain immune homeostasis and controlled inflammatory signaling.

In COPD, airway dysbiosis is associated with persistent activation of innate immune pathways. Increased exposure to microbial-associated molecular patterns can engage pattern-recognition receptors, including Toll-like and NOD-like receptors, and may amplify NF-κB- and inflammasome-related signaling with production of inflammatory mediators such as CXCL8/IL-8 that increased neutrophil recruitment (Takeuchi and Akira, 2010). Neutrophils recruited into this environment release proteases, reactive oxygen species, and neutrophil extracellular traps, which can contribute to tissue injury, mucus hypersecretion, and impaired lung function; defective resolution may further prolong inflammation (Kayongo et al., 2023).

Another hallmark of COPD-related dysbiosis is alveolar macrophage dysfunction. Studies demonstrated impaired phagocytic capacity and altered activation/polarization states, may reduce bacterial clearance and thereby reinforce microbial persistence and chronic innate immune activation (Sandhu et al., 2024). Because much of the human evidence is associative, these mechanisms are best interpreted as mutually reinforcing host-microbiota interactions rather than a unidirectional effect of dysbiosis.

### Dysbiosis-driven altered adaptive immunity

Dysbiosis may also influence adaptive immunity, although direct causal evidence in human COPD remains more limited than evidence for adaptive immune abnormalities themselves. Persistent microbial antigen exposure could modify dendritic-cell activation and antigen presentation. COPD is characterized by altered adaptive immune profiles, including increased CD8+ cytotoxic T-cell infiltration and changes in CD4+ T-cell responses; lung microbiome composition has also been associated with host adaptive immune responses (Sze et al., 2015). Dysregulated host-microbiota signaling may therefore contribute to pro-inflammatory T-cell responses and impaired immune regulation. Altered regulatory T-cell activity and B-cell responses may further affect immune tolerance and mucosal defense. These observations support a microbiota-adaptive immunity interface in COPD, but they do not establish that dysbiosis is the sole or primary cause of these abnormalities (Sandhu et al., 2024; Sze et al., 2015).

Altogether, these maladaptive immune responses contribute to progressive lung tissue damage and airway remodeling.

### Persistent inflammation as an outcome of dysbiosis

Building on the evidence that dysbiosis profoundly alters immune function in COPD, it is equally important to consider how these microbial shifts translate into persistent and maladaptive inflammatory responses within the lung. Increasing evidence indicates that airway microbial communities actively interact with host immune pathways, and disruption of these interactions contributes to chronic inflammation and disease progression in COPD (Wang et al., 2016; Mammen and Sethi, 2016).

Innate and adaptive immunity are tightly intertwined with inflammatory processes, forming an integrated system that ensures host defense against invading pathogens while preserving tissue integrity. Under homeostatic conditions, immune activation and inflammation are tightly regulated and self-limiting. Indeed, immune activation triggers transient inflammation that resolves once the stimulus is cleared. However, when immune sensing or regulatory mechanisms are disrupted, inflammatory responses may become exaggerated or chronic, contributing to tissue damage and disease progression rather than protection (Brusselle et al., 2011; Barnes, 2016).

In COPD, this balance is frequently disrupted, resulting in sustained inflammation that contributes to progressive tissue damage. Persistent activation of inflammatory pathways involving airway epithelial cells, macrophages, neutrophils, and lymphocytes promotes oxidative stress, protease release, extracellular matrix degradation, and airway remodeling, ultimately contributing to progressive lung function decline (Brusselle et al., 2011; Barnes, 2016).

Growing evidence indicates that airway microbial dysbiosis is a major driver of this unwanted inflammatory state in COPD. Alterations in the composition and diversity of the lung microbiota, often characterized by an overrepresentation of potentially pathogenic bacteria and reduced microbial community stability, lead to continuous stimulation of immune sensing pathways (Wang et al., 2016; Mammen and Sethi, 2016).

This persistent microbial signaling, mediated through sustained activation of pattern recognition pathways, promotes excessive production of pro-inflammatory mediators, recruitment of innate immune cells, and amplification of inflammatory circuits (Takeuchi and Akira, 2010; Kayongo et al., 2023; Brusselle et al., 2011; Bals and Hiemstra, 2004). Importantly, dysbiosis-driven inflammation in COPD is not merely a byproduct of infection but represents an unwanted amplification of inflammatory signaling. Rather than supporting effective host defense and resolving infection, altered microbial-host interactions may promote ineffective microbial clearance and persistent immune activation, thereby exacerbating epithelial injury, mucus hypersecretion, and airway remodeling (Wang et al., 2016; Brusselle et al., 2011).

Thus, dysbiosis acts as a critical modulator of lung inflammation in COPD, linking microbial imbalance to chronic inflammatory burden and disease progression, even in the absence of overt pathogenic infection (Wang et al., 2016; Mammen and Sethi, 2016).

### Dysbiosis and compromised antimicrobial defense

In healthy airways, the microbiota acts as a “gatekeeper” that contributes to the regulation of lung antimicrobial effectors, including antimicrobial peptides (AMPs) such as cathelicidin (LL-37) and defensins, mucociliary clearance and epithelial barrier integrity. These mechanisms help prevent excessive microbial colonization while maintaining an appropriate immune tone, allowing rapid responses to infection and limiting unnecessary inflammation (Bals and Hiemstra, 2004; Dickson et al., 2014).

In COPD, airway dysbiosis occurs alongside chronic epithelial injury, cigarette smoke exposure, and immune dysregulation, disrupting the reciprocal interactions between microbial communities and host antimicrobial defenses. Altered microbial-host signaling is thought to contribute to impaired expression and function of antimicrobial defense mechanisms, including AMPs such as defensins, LL-37, and secretory leukocyte protease inhibitor (SLPI), although these defects are also influenced by chronic inflammation and epithelial dysfunction (Mammen and Sethi, 2016; Bals et al., 1998). Consequently, compromised antimicrobial defenses create a permissive environment for persistent microbial colonization and increase susceptibility to infections and exacerbations (Wang et al., 2016; Sethi et al., 2006).

The impact of airway dysbiosis in COPD reflects an intricate and reciprocal relationship between microbial communities, host antimicrobial defenses, and immune/inflammatory responses. While dysbiosis is increasingly recognized for its role in amplifying inflammation and shaping maladaptive immune responses, it also interacts closely with intrinsic antimicrobial defense mechanisms that are essential for maintaining lung homeostasis (Mammen and Sethi, 2016; Dickson et al., 2014).

Such impairment of antimicrobial defenses is tightly linked to immune dysregulation and chronic inflammation in COPD, as LL-37 and defensins function not only as direct antimicrobial molecules but also as immunomodulatory mediators capable of influencing epithelial signaling, leukocyte recruitment, and inflammatory responses (Bals and Hiemstra, 2004; Nijnik and Hancock, 2009). Briefly, dysbiotic microbial communities may promote sustained activation of innate immune pathways, including pattern recognition receptor signaling, leading to excessive cytokine and chemokine production. However, this heightened inflammatory state does not necessarily translate into effective microbial clearance. Instead, persistent inflammation further damages epithelial cells and disrupts host defense mechanisms, creating a vicious cycle in which defective antimicrobial protection and aberrant immune activation reinforce one another (Mammen and Sethi, 2016; Brusselle et al., 2011; Barnes, 2016).

Moreover, inhaled corticosteroid therapy and repeated antibiotic exposure, frequently required during COPD exacerbations, can further alter airway microbial communities and may contribute to impaired host-microbiota equilibrium and reduced antimicrobial resilience (Wang et al., 2016; Mammen and Sethi, 2016).

Together, these processes highlight antimicrobial defense as a critical and distinct dimension of dysbiosis-associated pathology in COPD. Beyond modulating immune responses and inflammation, dysbiosis may contribute to impaired airway antimicrobial capacity, while defective host defenses simultaneously favor microbial persistence, thereby perpetuating colonization, exacerbations, and disease progression (Wang et al., 2016; Mammen and Sethi, 2016; Sethi et al., 2006).

### Dysbiosis and altered barrier integrity in COPD

The airway epithelial barrier, comprising intercellular junctions, the mucus layer, and local immune defenses, provides a critical interface between the respiratory microbiota and the host and constitutes a first line of defense against inhaled agents and microorganisms (Raby et al., 2023).

In healthy lungs, reciprocal interactions between the microbiota and airway epithelium contribute to epithelial homeostasis, barrier integrity, and local immune regulation. In turn, mucus production, antimicrobial defenses, and mucociliary clearance help shape the respiratory microbiota, maintaining a balanced host-microbiota interface (Raby et al., 2023; Bals and Hiemstra, 2004; Dickson et al., 2015).

In COPD, disruption of this finely tuned interplay contributes to a disease-associated vicious cycle. While airway dysbiosis is documented in COPD, microbial alterations and epithelial barrier dysfunction appear to interact bidirectionally and may mutually amplify disease progression (Raby et al., 2023; Mammen and Sethi, 2016). On one hand, cigarette smoke and air pollution induce structural and functional disruption of epithelial tight and adherens junctions, thereby increasing barrier permeability and facilitating exposure to microbial products, sustained immune activation, and chronic inflammation (Aghapour et al., 2022). Studies have described altered expression and redistribution of junctional proteins, including E-cadherin and ZO-1, together with abnormal epithelial remodeling and impaired repair, all of which characterize the airway epithelium in COPD (Zheng et al., 2025). On the other hand, microbial dysbiosis may alter host-microbiota interactions at the epithelial level, contributing to increased release of pro-inflammatory mediators, oxidative stress, and impaired epithelial repair mechanisms, thereby exacerbating inflammatory responses and compromising the ability to maintain an effective barrier (Bals and Hiemstra, 2004, Li et al., 2024). Mechanistically, increased epithelial permeability may facilitate greater exposure of subepithelial tissues and immune cells to microorganisms and their products, including lipopolysaccharide and other microbial-associated molecular patterns, thereby further fueling inflammation and disrupting local homeostasis. In this context, altered microbial signals may drive activation of pattern-recognition pathways on epithelial and immune cells, reinforcing the production of pro-inflammatory cytokines and chemokines and contributing to oxidative and proteolytic injury that can further compromise intercellular junctions (Wang et al., 2016). The association between airway microbial alterations and activation of Toll-like receptor and TNF-related inflammatory pathways has also been observed in patients with acute exacerbations of COPD, although causal relationships remain to be established (Li et al., 2024).

Moreover, dysbiosis in COPD is associated with alterations in mucus production and airway mucosal function, contributing to mucus hypersecretion and impaired mucociliary clearance. These changes can compromise the physical barrier and create conditions favorable to bacterial persistence, particularly during exacerbations (Wang et al., 2016; Raby et al., 2023). Rather than assuming that pathogen-dominated communities directly downregulate epithelial antimicrobial responses, it may be more accurate to state that persistent microbial exposure and epithelial stress can further impair epithelial antimicrobial and barrier functions, thereby weakening barrier resilience.

Impaired barrier integrity also affects epithelial-immune communication. Increased permeability enhances exposure of subepithelial immune cells to microbial products, amplifying inflammatory signaling and potentially skewing immune responses toward chronic, non-resolving inflammation (Li et al., 2024). This persistent inflammatory milieu further inhibits epithelial regeneration and promotes abnormal remodeling, contributing to airway wall thickening and progressive loss of functional airway surface (Raby et al., 2023; Barnes, 2016). Evidence has also suggested that epithelial barrier integrity in COPD may be influenced by gut microbiota dysbiosis, which may contribute to systemic inflammation and altered immune signaling through the gut-lung axis and indirectly affect airway epithelial function and repair capacity (Wang et al., 2023; Song et al., 2024). A working hypothesis is that circulating microbial metabolites and inflammatory mediators derived from the gut may influence pulmonary immune and epithelial responses, particularly in advanced disease; however, direct evidence linking gut-derived microbial metabolites to impaired airway epithelial repair in COPD remains limited and requires further investigation (Song et al., 2024).

Thus, barrier dysfunction in COPD is both a potential driver and a consequence of airway dysbiosis, contributing to a self-reinforcing cycle of persistent inflammation, defective epithelial repair, and progressive airflow obstruction. A useful conceptual framework is that lung dysbiosis and epithelial barrier disruption in COPD are not isolated phenomena but rather interconnected processes that can reinforce chronic inflammation, defective epithelial repair, microbial persistence, and progressive airway remodeling (Raby et al., 2023; Mammen and Sethi, 2016).

### Gut-Lung axis and immune responses

Beyond the lung, the gut microbiota represents a critical pathway that exerts systemic immune modulation, linking dysbiosis to adaptive and innate immune dysfunction in COPD. Normal gut microbes produce metabolites, particularly short-chain fatty acids (SCFAs), that support mucosal integrity and play a critical role in regulating immune cell differentiation and function. Thus, SCFAs can promote Treg differentiation, suppress excessive inflammatory signaling, and maintain epithelial barrier integrity (Maruyama et al., 2023; Ni et al., 2026).

In COPD, alterations in the gut microbiota have been associated with reduced levels of short-chain fatty acids (SCFAs) and an imbalance in key bacterial taxa. These processes may impact both innate immune cells (e.g., macrophages, neutrophils) and adaptive immune populations (e.g., Tregs, effector T cells), thereby amplifying pulmonary inflammation through systemic immune priming (Li et al., 2021; Zhu et al., 2025). For instance, changes in microbial metabolites can influence the differentiation and function of immune cells, including regulatory T cells (Tregs) and other immune populations, further dysregulating immune homeostasis across mucosal sites (Maruyama et al., 2023; Ni et al., 2026). Also, gut dysbiosis and associated alterations in intestinal barrier function may facilitate the systemic exposure to microbial products, which can contribute to systemic immune activation and potentially influence pulmonary immune responses (Ni et al., 2026).

In support of these observations and as mentioned above, experimental studies in which fecal microbiota from COPD patients were transplanted into mice provide evidence that COPD-associated gut dysbiosis can influence lung inflammatory responses. In such human-to-mouse fecal microbiota transplantation study, gut microbiota from COPD patients were associated with lower SCFA levels and induced increased lung inflammation in recipient mice; when combined with biomass-fuel smoke exposure, this was associated with accelerated decline in lung function, emphysematous changes, airway remodeling, and mucus hypersecretion (Li et al., 2021). These findings highlight the systemic nature of immune dysregulation in COPD and underscore the importance of microbiota-driven immune crosstalk (Maruyama et al., 2023; Li et al., 2021).

### Emerging mechanisms linking lung microbiota to COPD pathogenesis

While the immune, epithelial, inflammatory, and antimicrobial consequences of lung dysbiosis provide critical insight into COPD pathogenesis, a broader perspective that captures the complexity of this pathology is required. In recent years, additional dimensions have indeed emerged that further influence disease progression and clinical outcomes (Millares and Monso, 2022).

Beyond species shifts, dysbiosis is increasingly recognized as a functional alteration, in that changes in microbial metabolic activity and bioactive products can modulate airway inflammatory and immune signaling and epithelial responses and repair. Indeed, longitudinal microbiome studies have identified changes in predicted microbial functions during COPD exacerbations, including alterations in metabolic pathways and a reduction in microbial functions potentially associated with antimicrobial and anti-inflammatory activity (Wang et al., 2016; Huang et al., 2014). Lung microbiota composition also exhibits temporal variability during disease progression and acute exacerbations, with greater microbiome instability observed in some patients during exacerbations and in association with more severe clinical outcomes (Wang et al., 2018; Keir et al., 2021). Importantly, commonly used COPD therapies, including inhaled corticosteroids and antibiotics, interact with the lung microbiota, influencing microbial composition and potentially modifying host immunity, inflammation, and infection risk (Keir et al., 2021; Dy and Sethi, 2016; Richardson et al., 2025). As there are various COPD inflammatory endotypes, microbiota-host interactions likely vary across patients, reflecting disease-specific microbial signatures rather than a uniform dysbiotic state (Millares and Monso, 2022; Wang et al., 2018). Airway remodeling, mucus plugging, and tissue destruction, as observed in emphysema, may further create distinct ecological conditions that influence microbial persistence and dysbiosis (Wang et al., 2021; Millares and Monso, 2022).

### Determinants of lung microbiota composition in COPD

Lung microbiota composition reflects a finely regulated and dynamic balance between microbial entry, clearance, and niche-specific growth. These processes are governed by host-related factors, environmental exposures, and therapeutic interventions, collectively determining microbial community structure in health and disease (Dickson et al., 2015; Segal et al., 2016). In COPD, lung microbiota composition corresponds to the net result of interactions between such processes that generate conditions favoring dysbiosis (Pragman et al., 2012).

Among the primary determinants of lung microbiota composition are host genetic and mucosal-barrier factors. COPD-associated genetic variation in pathways involved in innate immune sensing, mucus production, and mucociliary clearance may influence host-microbiota interactions and the inflammatory consequences of microbial exposure. For example, mucins such as MUC5B and MUC5AC are essential components of the airway mucus barrier and mucociliary defense, while alterations in their concentration and composition can impair mucus transport and contribute to microbial retention and airway disease. Increased airway mucin concentrations, particularly MUC5AC, have been associated with COPD severity, exacerbation risk, and lung-function decline (Radicioni et al., 2021; Pincikova et al., 2024).

Environmental exposures are also critical determinants. Cigarette smoke, air pollution, and inhaled particulates modify the airway microenvironment and host defense mechanisms, potentially altering microbial persistence and community structure. Consistent with this, experimental and clinical evidence indicates that particulate matter can impair airway antibacterial defense and epithelial barrier integrity, thereby increasing susceptibility to microbial invasion and potentially contributing to dysbiosis (Chen et al., 2018; Liu et al., 2019).

Therapeutic pressures are key determinants of lung microbiota composition in COPD as well. Antibiotic exposure, commonly used to treat acute exacerbations, can alter airway microbial communities and exert selective pressure on respiratory bacteria. In a recent observational study of patients with chronic lung disease, prophylactic antibiotic use was associated with reduced bacterial diversity and density, whereas antibiotic “rescue-pack” use was associated with antimicrobial resistance among pathogenic isolates (Rofael et al., 2023). Inhaled corticosteroids (ICS), while beneficial in selected patients, can also influence the airway microbiome by modifying local immune responses and microbial ecology, with studies reporting increased bacterial load and enrichment of potentially pathogenic taxa in some ICS-treated patients (Pincikova et al., 2024). Importantly, these effects appear to depend on treatment intensity, COPD phenotype, and baseline microbiome composition rather than representing a uniform effect of all ICS regimens.

Together, these interventions can reshape the airway microbial ecosystem, promoting dysbiosis that may contribute to disease progression, exacerbation risk, and altered treatment responsiveness (Pincikova et al., 2024; Rofael et al., 2023). Systemic and lifestyle factors, including age, diet, and comorbidities, also influence lung microbiota composition. As mentioned above, alterations in gut microbial metabolites and immune signaling can modulate pulmonary immune responses and indirectly affect lung microbial ecology (Li et al., 2021; Li et al., 2023).

Once established, lung dysbiosis can perpetuate a vicious cycle of inflammation and microbial imbalance. Inflammatory conditions can alter the airway microenvironment, including mucus properties, epithelial integrity, and nutrient availability, thereby creating ecological conditions that may further favor dysbiotic microbial communities and contribute to the progression of chronic lung disease such as COPD (Dickson et al., 2015). Importantly, recent longitudinal evidence indicates that airway dysbiosis characterized by enrichment of opportunistic pathogenic taxa is associated with accelerated FEV₁ decline, supporting the possibility that alterations in the airway microbial ecosystem are not merely a consequence of established disease but may also participate in disease progression (Liang et al., 2023).

## Mucus-microbiota interactions in COPD

Airway mucus represents an important ecological interface between the host epithelium and the respiratory microbiota in COPD. Alterations in MUC5AC and MUC5B expression, mucus concentration, and rheology can impair mucociliary clearance and create spatially heterogeneous niches that favor microbial persistence. Mucus and microbial composition also appear to influence one another: nontypeable *Haemophilus influenzae* can stimulate MUC5AC transcription while recent clinical data link airway SCFAs, microbial composition, and mucus plugging. Thus, mucus hypersecretion and microbial dysbiosis may form a reinforcing loop in which impaired clearance favors microbial persistence, while microbial products and inflammatory responses further alter mucus production and its properties (Radicioni et al., 2021; Pincikova et al., 2024; Wang et al., 2002; Tanabe et al., 2025).

### Species composition of the lung microbiota in health and COPD

Given the dual role of the lung microbiota as both a contributor to pulmonary homeostasis and a potential driver of inflammation and disease, it is essential to identify the microbial species involved in health and COPD.

Advances in culture-independent sequencing have established that the lower respiratory tract harbors a low-biomass but diverse microbiota, challenging again the long-held view of lung sterility. In healthy individuals, the lung microbiota is shaped by a balance between microbial immigration from the upper airway, primarily through microaspiration, and elimination through host immune defenses and mucociliary clearance, resulting in a relatively dynamic community (Segal et al., 2013; Natalini et al., 2023). Healthy lungs are dominated by bacteria from the phyla Firmicutes, Bacteroidetes, Proteobacteria, and Actinobacteria, with frequent detection of oral-associated commensal genera such as *Prevotella, Veillonella, Streptococcus, Neisseria, Rothia, and Actinomyces* (Table 1) (Charlson et al., 2011; Segal and Blaser, 2014). Among these, *Prevotella* and *Veillonella* are frequently reported as abundant genera, although their functional significance in healthy lungs remains incompletely defined. Importantly, enrichment of these upper-airway-associated taxa is not necessarily associated with reduced inflammation; in healthy individuals, greater representation of supraglottic taxa has also been associated with increased pulmonary inflammatory markers, including lymphocytes and neutrophils (Segal and Blaser, 2014; Dickson et al., 2015).

**Table 1:** Summary of reported microbiota profiles in healthy and COPD lungs.

Feature	Healthy lungs	COPD lungs
**Community organization**	Low-biomass, dynamic communities shaped largely by immigration from the upper airways and elimination through mucociliary and immune clearance	Altered community composition and dysbiosis; reduced diversity is reported in many studies but is not consistent across cohorts and sampling methods
**Predominant bacterial groups**	Firmicutes, Bacteroidetes/Bacteroidota, Proteobacteria and Actinobacteria	Proteobacteria enrichment particularly in dysbiotic and exacerbation-associated profiles
**Frequently detected genera**	Prevotella, Veillonella, Streptococcus, Neisseria, Rothia and Actinomyces	Enrichment of Haemophilus, Moraxella, and particularly in advanced disease, Pseudomonas
**Representative COPD-associated species**	No single species defines a healthy lung microbiota	Haemophilus influenzae (particularly non-typeable strains), Moraxella catarrhalis, Streptococcus pneumoniaeand Pseudomonas aeruginosa
**Oral-associated taxa**	Prevotella and Veillonella are frequently abundant; enrichment with supraglottic taxa can nevertheless be associated with pulmonary inflammatory responses	Relative depletion of Prevotella, Actinomyces and Atopobium has been reported, whereas changes in Veillonellaare inconsistent across populations and disease states
**Exacerbations**	Community composition reflects a dynamic equilibrium between immigration and clearance	Exacerbations can be accompanied by community shifts and enrichment or dominance of Moraxella, Haemophilus, Pseudomonas and other Proteobacteria

In contrast, the lung microbiota in COPD is associated with marked alterations in composition, commonly described as dysbiosis. Comparative studies with healthy controls using sputum, bronchoalveolar lavage, and bronchial brush samples have demonstrated alterations in community composition and, in many studies, reduced microbial diversity together with enrichment of potentially pathogenic taxa, particularly within the Proteobacteria phylum in COPD airways (Ramsheh et al., 2021; Kou et al., 2024). However, the evidence for uniformly reduced α-diversity is not entirely consistent across studies and sampling methods, emphasizing the heterogeneity of COPD-associated microbiome profiles (Kou et al., 2024).

Microbial composition in COPD is not static but varies with disease severity and exacerbation status. At the species and genus level, *Haemophilus influenzae* (especially non-typeable strains), *Moraxella catarrhalis*, and *Streptococcus pneumoniae* are frequently detected, and, in more advanced disease, *Pseudomonas aeruginosa* may become particularly prominent (Wang et al., 2020; Bouquet et al., 2020). These shifts are accentuated during acute exacerbations and may occur in conjunction with viral infections. Longitudinal multicentre data indicate that *Moraxella* and *Haemophilus* are more likely to dominate during exacerbations, while *Pseudomonas* and other Proteobacteria are associated with exacerbation frequency (Bouquet et al., 2020). Similarly, longitudinal microbiome studies have demonstrated enrichment of Proteobacteria, including H. influenzae and other potentially pathogenic taxa, during exacerbations (Huang et al., 2014; Xue et al., 2023).

### Changes in the lung microbiota during stable COPD, exacerbations, and recovery

Microbiota patterns should be interpreted differently across clinical states. Some stable COPD cohorts show reduced diversity and enrichment of Proteobacteria or Haemophilus, but these features are heterogeneous and are not universal across patients. During exacerbations, at least three overlapping biological patterns can be distinguished: bacterial exacerbations with increased bacterial burden or dominance of taxa such as Haemophilus and Moraxella; virus-associated exacerbations in which respiratory viral infection may precede or accompany secondary bacterial shifts; and eosinophilic exacerbations in which major bacterial community changes may be less pronounced. Longitudinal AERIS, COPDMAP, and multicohort analyses further show that microbiome instability can increase during exacerbations and that bacterial and eosinophilic exacerbation phenotypes may recur within individuals. Recovery after treatment may partially restore the pre-exacerbation community, but antibiotics and corticosteroids can themselves alter this trajectory. These observations argue against treating “the exacerbation microbiome” as a single state and instead support a framework of stable COPD-Exacerbation-recovery (Wang et al., 2021; Wang et al., 2018; Bouquet et al., 2020; Mayhew et al., 2018; Huang et al., 2026).

These microbial shifts have been associated with heightened airway inflammation, including neutrophilic inflammatory responses, increased symptom burden, and worse clinical outcomes. Importantly, however, the relationship between individual taxa and clinical outcomes is complex and depends on microbial abundance, community context, host inflammatory phenotype, and disease state (Ramsheh et al., 2021; Dicker et al., 2021). Interestingly, there is evidence for a relative depletion of several oral-associated genera, including *Prevotella, Actinomyces*, and *Atopobium*, in COPD compared with healthy controls, although the direction and magnitude of changes in *Veillonella* vary according to disease state and study population (Kou et al., 2024).

These findings should nevertheless be interpreted cautiously because species-level identification and between-study comparisons remain constrained by sampling and analytical factors. These methodological issues are considered in a dedicated paragraph below (Caverly et al., 2019; Salter et al., 2014; Fierer et al., 2025).

Many studies are also cross-sectional and variably control for smoking status, inhaled corticosteroid exposure, antibiotic use, and disease severity. Longitudinal and multicentre studies increasingly demonstrate dynamic changes across clinical states, supporting repeated sampling and standardized approaches when evaluating microbial trajectories and potential causal relationships (Wang et al., 2018; Bouquet et al., 2020).

Taken together, these findings underscore the lung microbiota as a double-edged sword, whereby relatively commensal-dominated communities may contribute to airway homeostasis, while dysbiotic, pathogen-enriched profiles can contribute to chronic inflammation and disease progression in COPD. However, the functional consequences of individual taxa are highly context-dependent, and the distinction between “commensal” and “pathogenic” communities should therefore not be regarded as absolute.

### Methodological considerations in respiratory microbiome studies

When interpreting lower-airway microbiome studies, attention must be paid to sampling modality, microbial biomass, sequencing resolution, and analytical pipelines. While sputum is relatively accessible, it can however contain substantial upper airway material. Similarly, bronchoalveolar lavage samples allow access to the lower respiratory tract but remain low-biomass. They may be influenced by upper airway carryover and dilution, as well. Bronchial brushings permit protected sampling and provide more localized information. But, they are invasive and sample only restricted niches. These differences can impact observed community composition and diversity. Of relevance, low biomass also increases the relative contribution of DNA from extraction kits, reagents, and laboratory environments. As such, extraction blanks, reagent controls, negative controls, and contamination-aware filtering are essential (Segal et al., 2013; Natalini et al., 2023; Salter et al., 2014; Fierer et al., 2025).

Other considerations concern molecular issues. 16S rRNA gene sequencing remains useful for broad taxonomic profiling but provides limited species- and strain-level resolution and relies on relative-abundance measures. Shotgun metagenomics can improve taxonomic resolution and characterize genes and metabolic potential, whereas metatranscriptomics provides information on transcriptionally active functions but remains particularly sensitive to RNA quality and low biomass. Consequently, differences in alpha- or beta-diversity, differential abundance, or inferred function depend on sampling, sequencing platform, reference database, contaminant removal, normalization, and statistical pipeline. Cross-study comparisons therefore require standardized protocols and transparent reporting instead of assuming that the observed differences represent biological variation (Yan et al., 2022; Caverly et al., 2019; ; Salter et al., 2014; Fierer et al., 2025; Wang et al., 2020).

### Comparison with other chronic pulmonary diseases

Several features of the COPD airway microbiota are shared with other chronic pulmonary diseases and should not be considered COPD-specific. Asthma, bronchiectasis, and cystic fibrosis can all show altered diversity, Proteobacteria enrichment, or pathogen-associated community states, although the dominant organisms, inflammatory endotypes, mucus abnormalities, and clinical consequences differ. Cross-disease meta-analyses identify disease-specific taxonomic and functional signatures, while recent lower-airway sequencing data also show both shared and disease-enriched microbial networks between asthma and COPD. In idiopathic pulmonary fibrosis, increased bacterial burden and altered communities have likewise been associated with disease progression, illustrating that microbiome-host interactions extend beyond obstructive lung disease. These comparisons support the view that COPD is characterized not by a unique “COPD microbiome,” but by disease- and endotype-specific combinations of microbial structure, function, host response, and airway environment (Huang et al., 2026; Caverly et al., 2019; Ghosh and Saha, 2022; Molyneaux et al., 2014; Carrera-Salinas et al., 2023).

### Dysbiosis in COPD: a cause or consequence?

Whether dysbiosis initiates COPD, emerges as a consequence of the diseased airway, or performs both roles at different stages remains unresolved. Current evidence favors a bidirectional model: smoking, airway injury, inflammation, structural remodeling, and therapeutic exposure can reshape microbial ecology, while established dysbiosis may subsequently amplify host inflammation, alter microbial metabolism, and impair clearance (Liang et al., 2023; Dicker et al., 2021). This reciprocal relationship is central to the double-edged-sword concept because the biological effect of the microbiota depends on the host and ecological context in which it is embedded.

On one hand, cigarette smoking, the predominant cause of COPD, directly perturbs both lung and gut microbial communities. Smoking modifies the local microenvironment, resulting in increased oxidative stress and altered mucosal barrier integrity, which can disrupt microbial balance in both respiratory and intestinal niches (Laiman et al., 2024). As such, dysbiosis could be secondary to COPD and its major environmental risk factors. Experimental and observational studies indicate that cigarette smoke itself can alter lung and intestinal microbial composition, supporting the concept that some microbial alterations may arise independently of established COPD (Laiman et al., 2024).

Interestingly, airway microbiome alterations have been reported in COPD compared with smokers without COPD, suggesting that dysbiosis is not simply a consequence of smoking exposure alone and may also reflect chronic lung injury and disease-related host responses (Haldar et al., 2020; Erb-Downward et al., 2011). Furthermore, systemic effects of COPD such as hypoxia, oxidative stress, nutritional abnormalities, recurrent infections, and pharmacological treatments could themselves modify microbiota composition, supporting the possibility that dysbiosis may also emerge or intensify during disease progression (Wang et al., 2020; Richardson et al., 2025).

On the other hand, there is also evidence supporting a contributory role of dysbiosis in COPD pathogenesis. As mentioned above, experimental studies have shown that transplantation of fecal microbiota from COPD patients into mice can exacerbate pulmonary inflammation and, in the context of biomass-fuel smoke exposure, accelerate lung-function decline, emphysematous changes, airway remodeling, and mucus hypersecretion, implying that gut microbial alterations can influence pulmonary immune responses and tissue injury (Li et al., 2021). This suggests that dysbiotic communities may skew host immune signaling, increase pro-inflammatory responses, and alter microbial metabolic outputs such as short-chain fatty acids, which have important immunoregulatory effects at distant mucosal sites (Li et al., 2021; Song et al., 2023). These observations align with the concept of the gut-lung axis, whereby microbial metabolites and immune mediators produced in the gut can modulate lung inflammation and susceptibility to injury, potentially contributing to COPD progression.

Most human studies linking dysbiosis to COPD severity, exacerbations, and immune alterations remain observational and cannot establish causality. A plausible model is that COPD-related exposures and structural changes initially reshape the airway ecosystem, after which dysbiosis feeds back to sustain inflammation and immune dysregulation (Wang et al., 2020; Dicker et al., 2021). Longitudinal evidence that baseline airway dysbiosis is associated with accelerated FEV1 decline, together with experimental evidence implicating *Staphylococcus aureus* in impaired lung function, strengthens the possibility that microbial alterations can act as mediators as well as markers of disease (Wang et al., 2020). Nevertheless, longitudinal and mechanistic studies are still required to determine when microbial changes precede disease progression and when they primarily reflect established pathology.

### Evidence supporting the bidirectional model

The evidence for the two directions of the dysbiosis-COPD relationship is not equivalent. Evidence that COPD-related exposures and treatments reshape the airway microbiota is relatively strong and includes cross-sectional comparisons, longitudinal cohorts, and intervention studies showing microbiome changes after inhaled corticosteroids or antibiotics. Evidence that microbial alterations contribute to COPD progression is more limited in humans: longitudinal studies associate baseline dysbiosis with subsequent lung-function decline, while experimental airway and gut-lung studies in animals provide mechanistic support. Fecal microbiota transplantation experiments are particularly informative because they demonstrate that COPD-associated gut communities can modify pulmonary inflammation or COPD-like features in mice, but they do not establish causality in humans. Donor selection, pooled samples, host species differences and experimental smoke exposures limit direct translation. Accordingly, current evidence strongly supports an association between COPD and its treatments and changes in the lung microbiota. Evidence that the microbiota contributes to disease development and progression is moderate, based on longitudinal human studies and experimental research. However, definitive evidence establishing a causal role for the microbiota in human COPD remains insufficient (Wang et al., 2020; Yan et al., 2022; Sulaiman et al., 2023; Li et al., 2021; Liang et al., 2023).

## Exploratory prognostic and theragnostic approaches

COPD is a clinically heterogeneous disorder characterized by variable trajectories of progression, exacerbation risk, and response to therapy, as recognized in current international guidelines. Despite substantial advances in the understanding of airway inflammation and structural remodeling and guideline-driven management strategies, current tools remain insufficient to accurately predict individual disease trajectories or guide fully personalized treatment strategies. In this context, the recognition of lung microbiota as an active regulator of airway homeostasis and inflammation has opened new avenues for prognostic and theragnostic exploration (Luo et al., 2024; Yu et al., 2023).

Lung dysbiosis is increasingly viewed not simply as a consequence of disease but as a dynamic component of COPD pathogenesis, integrating host susceptibility, environmental exposures, and therapeutic-mediated pressures. By modulating inflammatory tone, epithelial responses, and microbial resilience, dysbiosis may influence disease progression and exacerbation risk, a concept that is supported by longitudinal studies including that in an independent cohort (Liang et al., 2023).

As such, microbiota-associated alterations may capture disease-relevant information not accessible through conventional clinical or physiological parameters and may complement established parameters to refine risk stratification and disease classification. Indeed, airway microbial profiles have been associated with COPD severity, exacerbation frequency, inflammatory phenotypes, and clinical outcomes, supporting their potential value as complementary biomarkers rather than replacements for established clinical measures (Luo et al., 2024; Yu et al., 2023). Together, these elements position lung dysbiosis as a promising source of novel biomarkers and/or as a means of identifying biological pathways that could be targeted therapeutically. Recent exploratory strategies leveraging high-resolution omics approaches, particularly when combined with immune and epithelial profiling, provide a framework for defining functional biomarkers with prognostic and theragnostic relevance (Liang et al., 2023; Leitao Filho et al., 2021).

These approaches are well positioned to inform guideline-relevant outcomes, including exacerbation risk, treatment responsiveness, and disease stability. Importantly, lung dysbiosis could represent a potentially modifiable component of COPD, opening perspectives for interventional strategies aimed at restoring microbial balance or limiting its pathogenic consequences. Proof-of-concept experimental evidence is particularly intriguing in this regard: in a recent study, depletion of Staphylococcus aureus using bacteriophages attenuated microbiome-associated lung-function decline in emphysema mice, suggesting that specific microbial taxa or microbial functions could eventually represent therapeutic targets (Liang et al., 2023).

Also, therapeutic modulation of the airway microbiota, including antibiotics and corticosteroids, may not only influence disease outcomes but also reshape microbial ecosystems in ways that impact treatment efficacy and resistance. Longitudinal studies have demonstrated that antibiotic and corticosteroid treatment during COPD exacerbations can produce distinct and sometimes opposing changes in airway microbial composition (Huang et al., 2014). Randomized clinical trials have further shown that inhaled corticosteroid-containing regimens, particularly fluticasone-based therapy, can modify the airway microbiome (Richardson et al., 2025; Mayhew et al., 2018). Understanding these interactions is therefore essential to optimize current treatment strategies and to identify patient subgroups most likely to benefit from microbiota-informed interventions.

Importantly, however, microbiome-based prognostic and theragnostic approaches remain exploratory. Differences in sampling methods, sequencing platforms, analytical pipelines, microbial biomass, and patient characteristics currently limit cross-study comparability and preclude routine clinical implementation. Prospective longitudinal validation and standardized methodologies will therefore be required before microbiome-derived signatures can be incorporated into clinical risk prediction or treatment selection (Caverly et al., 2019; Yu et al., 2023).

Building on this framework, the following sections will focus on omics-driven biomarker discovery and interventional studies, examining how microbiota-associated signatures may refine guideline-based therapeutic decision-making and support the development of personalized treatment strategies in COPD.

### Maintenance of a stable and resilient lung microbiota

It is essential to emphasize that, prior to considering exploratory prognostic and theragnostic strategies targeting lung dysbiosis, the foremost objective should be preventive approaches aimed at preserving a stable lung microbiota while minimizing factors that may disrupt its ecological balance. Maintenance of microbial homeostasis within the lower respiratory tract is increasingly recognized as relevant to immune equilibrium, pathogen containment, and inflammatory tone, whereas disturbances in community structure and temporal stability have been associated with exacerbations and disease instability in COPD (Wang et al., 2021). Longitudinal studies have shown that the airway microbiome is generally more stable during clinical stability and becomes more dynamically altered during exacerbations, particularly in patients with frequent exacerbations (Wang et al., 2021). Moreover, Moreover, therapeutic pressures, including antibiotics and inhaled corticosteroids, can alter airway microbial composition, although the magnitude and direction of these effects depend on the treatment, disease phenotype, and underlying microbial community (Richardson et al., 2025; Rofael et al., 2023; Mayhew et al., 2018).

Taken together, these observations suggest that preserving microbial stability and ecological resilience may represent a relevant preventive objective in COPD, although this concept remains to be formally validated in interventional studies. Because guideline-recommended therapies can themselves alter the airway microbiota, a realistic goal is not necessarily to restore an idealized “eubiotic” composition, but to preserve or recover functional resilience while maintaining treatments with established clinical benefit. This approach requires defining which microbial functions or interaction networks are clinically meaningful rather than assuming that normalization of taxonomic composition is always desirable.

### Prognostic role of lung dysbiosis in COPD

Microbial instability and loss of community resilience have been associated with worse clinical outcomes in COPD. As such, lung dysbiosis is increasingly recognized as a dynamic marker of disease activity and clinical phenotype in COPD, reflecting the cumulative impact of host susceptibility, environmental exposures, and therapeutic treatments. Airway microbial communities evolve over time and may therefore capture longitudinal information relevant to disease progression and exacerbation risk. This positions dysbiosis as a potential prognostic dimension complementary to established parameters such as lung function decline, symptom burden, and exacerbation history (Wang et al., 2016; Pragman et al., 2024). In the AERIS longitudinal cohort, reduced temporal stability of the lung microbiome was more frequent during exacerbations and in patients with a higher exacerbation frequency, supporting the concept of microbial instability as a marker of disease activity (Salter et al., 2014). Similarly, in the COPDMAP cohort, dysbiosis occurring during exacerbations was associated with greater exacerbation severity, including larger declines in FEV₁ (Forced Expiratory Volume in one second) and FVC (Forced Vital Capacity) and greater worsening of COPD Assessment Test scores (Segal et al., 2016).

Rather than a consistent association between individual commensal taxa and future exacerbations, current evidence suggests that overall community structure and diversity may be more informative prognostic features. In a longitudinal case-control study, patients with the frequent-exacerbator phenotype had lower sputum microbiome α-diversity than infrequent exacerbators, together with greater airway inflammation (Li et al., 2022). Similarly, a metagenomic study of stable COPD patients found lower microbial diversity in frequent exacerbators and identified differences in the abundance of several taxa, including *Veillonella, Fusobacterium,* and *Prevotella*; importantly, these taxa were generally more abundant in infrequent rather than frequent exacerbators, arguing against their use as simple markers of poor prognosis (Chiu et al., 2022). Thus, microbial diversity and community organization may provide more robust prognostic information than the abundance of any single bacterial genus.

More compelling evidence links Proteobacteria-dominated airway communities, particularly those enriched in *Haemophilus,* with adverse clinical outcomes. In a large COPD cohort, Proteobacteria dominance and reduced microbiome diversity were associated with more severe airflow obstruction, more frequent exacerbations, lower FEV₁, and increased mortality; Proteobacteria-dominated profiles were associated with significantly higher mortality compared with Firmicutes-dominated or balanced microbiome profiles (Dicker et al., 2021). Integrated microbiome-proteomic analyses further linked Proteobacteria dominance to neutrophil-associated inflammatory pathways, providing biological plausibility for its association with adverse outcomes (Dicker et al., 2021).

Alterations in the gut microbiota may also provide prognostic information through the gut-lung axis. In a cohort of patients with COPD, greater disease severity was associated with alterations in gut microbial composition and reduced total fecal short-chain fatty acid (SCFA) concentrations, particularly in patients with more advanced disease (Li et al., 2021). Moreover, a one-year follow-up study found that changes in gut microbial composition were associated with subsequent lung-function decline; patients with declining lung function showed distinct gut microbiota profiles, including greater abundance of Firmicutes and lower abundance of Alloprevotella compared with controls (Jiang et al., 2024). These findings suggest that gut microbial composition and microbial metabolites may represent complementary biomarkers of systemic disease activity, although their independent predictive value for COPD progression remains to be established.

The potential importance of microbial metabolites is further supported by experimental evidence. SCFAs, particularly butyrate, can modulate pulmonary immune responses and airway inflammation, providing a plausible biological link between gut microbial metabolism and COPD outcomes (Yang et al., 2022). However, direct evidence that circulating or fecal SCFA concentrations independently predict future COPD exacerbations or lung-function decline in large prospective human cohorts remains limited. Therefore, SCFAs should currently be regarded as promising mechanistic and biomarker candidates rather than established prognostic biomarkers.

Moreover, integrating microbiota-related information may help refine patient stratification beyond current guideline-based classifications, particularly in patients with frequent exacerbations or rapid progression despite optimized therapy. The identification of distinct microbiome–inflammatory profiles, including Proteobacteria-dominated and more diverse microbial states, suggests that microbiome information could eventually contribute to COPD endotyping and individualized risk assessment (Dicker et al., 2021; Li et al., 2022). Nevertheless, prospective validation is required before microbiome-derived measures can be incorporated into routine prognostic models or clinical decision-making.

Taken together, current evidence supports lung dysbiosis as a potentially informative prognostic dimension in COPD, particularly when characterized by reduced community diversity, impaired temporal stability, or Proteobacteria/Haemophilus dominance. However, the prognostic value of individual taxa and gut-derived microbial metabolites remains less certain. Future longitudinal, multicentre studies integrating microbiome composition, microbial function, metabolomics, host inflammatory phenotypes, and clinical outcomes will be required to establish whether microbiome-derived signatures can improve prediction of exacerbations, lung-function decline, and mortality.

### Omics-based characterization of lung microbial signatures

High-throughput omics approaches have revolutionized research on lung dysbiosis by allowing more comprehensive and functional profiling of the microbial communities inhabiting the respiratory tract. Metagenomic and metatranscriptomic approaches extend beyond taxonomic profiling by providing information on microbial genes, metabolic pathways, and transcriptionally active microbial functions, thereby offering insights into how microbial activity may contribute to COPD pathophysiology (Ditz et al., 2022; Ren et al., 2018). Importantly, metatranscriptomic analyses have demonstrated that the lung microbiome is not simply a collection of detectable microbial taxa but includes a transcriptionally active component associated with host inflammatory status, highlighting the importance of microbial activity in addition to microbial presence (Yan et al., 2022).

These data provide critical insights into how, beyond species presence, microbial functional activity and metabolism may influence COPD outcomes. In particular, metagenomic and metatranscriptomic approaches can identify functional pathways potentially involved in microbial adaptation, metabolism, and host-microbe interactions (Ditz et al., 2022). When combined with metabolomic profiling and host-derived transcriptomic or proteomic data, multi-omic approaches enable an integrated view of host-microbiota interactions within COPD tissues. A particularly informative study integrated airway metagenomic, metabolomic, host transcriptomic, and proteomic data from 99 patients with COPD and 36 healthy individuals, identifying microbiome-metabolite-host interaction networks associated with neutrophilic and eosinophilic inflammatory endotypes. One proposed pathway involved altered microbial tryptophan metabolism, reduced indole-3-acetic acid, and perturbation of host IL-22 signaling and epithelial-cell apoptosis pathways (Wang et al., 2020).

Similarly, a multi-omic meta-analysis integrating airway microbiome datasets with host transcriptomic and metabolomic data identified functional microbial signatures associated with COPD and showed that predicted microbial metabolites could interact with COPD-associated host gene pathways. These microbiome-metabolite-host interactions were subsequently examined in an independent cohort in which airway metagenome, metabolome, and host transcriptome were simultaneously characterized, providing experimental support for the utility of integrated multi-omic approaches (Tiew et al., 2021). Such integrative frameworks are therefore particularly valuable for identifying candidate molecular signatures and biomarkers that capture both microbial dysbiosis and host response in a disease-relevant context, although their clinical utility still requires validation in larger longitudinal and independent cohorts.

### Microbiota-derived molecules as disease biomarkers

Specific microbial patterns or functional signatures offer promising opportunities as disease biomarkers. That is, if they are linked to defined disease aspects such as inflammatory phenotypes, exacerbation susceptibility, or treatment responses, they could become clinically meaningful for disease stratification. In COPD, several studies have identified airway microbial signatures associated with inflammatory phenotypes, exacerbation risk, mortality, and clinical disease states, supporting the potential use of microbiota-derived features for patient stratification (Dicker et al., 2021; Salter et al., 2014; Dang et al., 2022). These biomarkers could provide added value by identifying biological heterogeneity not captured by standard clinical measures such as spirometric or symptomatic evaluations. Importantly, multi-omic analyses have further suggested that functional microbial signatures and microbiota-derived metabolites may provide information beyond taxonomic composition, including potential links between microbial metabolic activity and host COPD-associated pathways (Wang et al., 2020).

However, their translation into clinical practice hinges on demonstrating reproducibility, temporal stability, external validation, and practical sampling strategies, given the well-documented technical and analytical variability in microbiota research. This is particularly relevant for low-biomass respiratory samples, in which contamination and differences in sampling, DNA extraction, sequencing, and analytical procedures can substantially influence the resulting microbial profiles (Fierer et al., 2025; Porcari et al., 2025). Accordingly, rigorous standardization of sampling procedures, analytical pipelines, and cross-cohort validation will be indispensable for the reliable prognostic and potentially theragnostic use of microbiota-associated signatures. Recent international consensus recommendations emphasize that the paucity of validated analytical methods, together with the need for standardized and clinically validated assays, remains a major barrier to the implementation of microbiota-based biomarkers in clinical practice (Rodriguez et al., 2025; Taherkhani et al., 2024).

### AI-driven insights into lung dysbiosis and COPD pathogenesis

Artificial intelligence (AI) offers a powerful framework to integrate high-dimensional microbial data with clinical and host variables to characterize the role of lung dysbiosis in COPD pathogenesis. Alterations in the respiratory microbiome, hence microbiota, have been consistently associated with clinical heterogeneity in COPD, including disease severity, symptom burden, inflammatory phenotype, and exacerbation frequency, suggesting that microbial communities may contribute to disease progression (Wang et al., 2021; Dicker et al., 2021). Machine learning models can leverage these complex patterns to identify microbial signatures associated with clinical phenotypes and outcomes. For example, supervised machine-learning approaches applied to sputum microbiome data have identified microbial features associated with mortality and other clinical characteristics in COPD (Liu and Zhao, 2026). More recently, a machine-learning model integrating clinical and airway microbiome features was able to predict the high-mucus-secretion phenotype in COPD, with *Haemophilus* and *Pseudomonas* abundance among the selected predictive features (Xiao et al., 2024).

Moreover, AI-assisted ecological and network analyses have revealed changes in microbial interactions and dysbiosis that align with COPD clinical stability and exacerbation phenotypes. Large-scale bacterial interactome analysis demonstrated reproducibly reduced antagonistic microbial interactions in COPD, both during clinical stability and exacerbations, and showed that these network alterations were associated with dyspnea, poorer lung function, neutrophilic inflammation, and increased exacerbation risk (Segal et al., 2017). Similarly, metabolic-network simulation combined with supervised machine learning identified alterations in microbial interactions and community structure associated with COPD exacerbation and clinical outcomes (Liu and Zhao, 2026). These findings suggest that microbial community structure and interspecies interactions may provide information beyond conventional measures of taxonomic abundance or diversity.

By combining advanced feature extraction, predictive modeling, network analysis, and multi-omics integration, AI approaches may uncover latent relationships between dysbiotic lung microbiota, host inflammatory responses, and COPD clinical phenotypes, thereby advancing biomarker discovery and patient stratification. Importantly, however, these approaches should currently be viewed primarily as tools for pattern recognition, prediction, and hypothesis generation, rather than as definitive evidence of causality. Their clinical and mechanistic value will require validation in larger, longitudinal, multicentre cohorts integrating microbiome, metabolomic, transcriptomic, immune, and clinical data.

### Potential treatment-response applications of lung dysbiosis

Beyond prognostic applications, lung dysbiosis holds promising potential for theragnostic applications, by informing disease status, treatment selection, and potentially predicting therapeutic response. In this regard, microbial signatures may help identify patients more likely to benefit from targeted interventions such as antibiotics or anti-inflammatory therapies and may also flag individuals at risk of adverse microbial shifts that could worsen outcomes. Although microbiome-guided treatment selection has not yet been validated in routine clinical practice, accumulating evidence indicates that airway microbial profiles are associated with COPD severity, exacerbation risk, and distinct inflammatory endotypes, supporting their potential use as biomarkers for patient stratification and treatment monitoring (Richardson et al., 2025; Tiew et al., 2021; Lim et al., 2023).

Chronic antibiotic treatment in frequent exacerbators has been shown to modify microbial communities and associated inflammatory mediators. In a randomized controlled study of patients with emphysema, azithromycin altered the lower-airway microbiome and metabolome and was associated with reductions in several inflammatory mediators, while bacterial metabolites induced by treatment were capable of attenuating inflammatory responses ex vivo (Lim et al., 2023). These findings provide mechanistic support for the concept that therapeutic effects of antibiotics may extend beyond direct antimicrobial activity, although the specific contribution of microbiome modulation to clinical benefit remains incompletely established. At the same time, prolonged azithromycin exposure can select for macrolide resistance and genetic adaptation in persistently colonizing Haemophilus strains illustrating the tension between antimicrobial benefit and ecological or evolutionary consequences (Carrera-Salinas et al., 2023; Pettigrew et al., 2016). Thus, microbiome restoration cannot simply be pursued independently of guideline-recommended antimicrobial or anti-inflammatory treatment. The objective should be to understand and, where possible, minimize clinically harmful ecological consequences while preserving proven treatment benefit.

In this context, dysbiosis serves both as a potential biomarker and a potential biological target. Regarding the latter, therapeutic alternatives could involve interventions using probiotics, prebiotics, and potentially fecal microbiota transplantation (FMT), careful use of antibiotics, or tracking changes in microbiota composition and function; all of which remain investigational approaches that may ultimately contribute to restoration of microbial and immune homeostasis. Current evidence for probiotics, prebiotics, dietary interventions, and FMT in COPD is still largely derived from preclinical studies and early clinical investigations, and their efficacy and safety require further validation before they can be considered established microbiome-targeted therapies (Yu et al., 2026; Hua et al., 2024).

Integrative theragnostic approaches that combine microbiota profiles with clinical and immunological data could support more personalized treatment strategies, particularly for patients with frequent exacerbations or suboptimal responses to standard therapies, aligning with the growing emphasis on precision medicine in COPD. Multi-omics analyses integrating the airway microbiota with microbial metabolites, host transcriptomic and proteomic profiles have already identified distinct host-microbe interactions associated with neutrophilic and eosinophilic inflammatory endotypes and have identified candidate microbiome-derived metabolites with potential therapeutic effects (Wang et al., 2020). Such integrated approaches may ultimately enable simultaneous patient stratification, prediction of therapeutic response, and monitoring of microbiome-related treatment effects, although prospective clinical validation is still required.

### Targeting microbiota to enhance therapeutic approaches

The recognition of lung dysbiosis as a potentially modifiable component of COPD has prompted growing interest in microbiota-targeted interventions. Increasing evidence indicates that airway dysbiosis, including enrichment of opportunistic pathogenic taxa, is associated with disease severity, exacerbations, and accelerated lung-function decline, supporting the concept that microbial imbalance may actively contribute to persistent inflammation rather than simply reflect structural lung damage (Liang et al., 2023; Kou et al., 2024). Understanding this relationship is key for future therapeutic strategies, including microbiota-based interventions.

In this context, strategies such as probiotics, prebiotics, and synbiotics aim to modulate microbial communities and host immune responses rather than simply suppress pathogenic taxa. Much of the current rationale is grounded in the gut-lung axis, whereby intestinal microbiota influences pulmonary immunity through systemic immune signaling and microbial metabolites (Li et al., 2021; Li et al., 2023). Although direct airway-targeted approaches remain technically challenging, modulation of the gut microbiota represents a potentially accessible adjunct strategy. Early clinical studies of oral probiotics in COPD have reported possible benefits, including delayed exacerbations or improvement in selected clinical and inflammatory outcomes, but these findings require confirmation in larger, well-controlled trials (Aghamohammadi et al., 2025; Kao et al., 2017). Regarding antibiotic stewardship, microbiome-informed and personalized antibiotic strategies remain investigational in COPD, with the potential to minimize unnecessary disruption of airway microbial communities while targeting clinically relevant pathogens.

Another alternative relies on fecal microbiota transplantation (FMT). FMT has demonstrated established efficacy in recurrent Clostridioides difficile infection (Minkoff et al., 2023; Cook et al., 2025) and has therefore been proposed as a broader means of restoring microbial diversity and immune homeostasis. However, its role in COPD remains speculative with experimental FMT studies in mice showing effects on pulmonary inflammation and COPD-like features, with no robust clinical evidence demonstrating efficacy in COPD patients. At present, FMT should therefore be regarded as an experimental, hypothesis-generating approach within the COPD field rather than a clinically validated intervention.

In addition to fecal microbiota transplantation (FMT), phage therapy offers a promising experimental approach to modulate lung dysbiosis in COPD. COPD is characterized by enrichment of opportunistic pathogens, among others, that are associated with inflammation and disease progression (Wang et al., 2020). Bacteriophages, viruses that specifically infect bacteria, can selectively reduce susceptible bacterial populations without broadly disrupting the bacterial community, potentially providing a more targeted alternative to conventional antibiotics. Recent evidence indicates that bacteriophage ecology is altered in COPD: phage diversity decreases with increasing disease severity, with disruption of phage-bacteria relationships particularly evident in patients with frequent exacerbations (Keir et al., 2020). Experimental models provide additional support for therapeutic potential: in an emphysema model, phage-mediated depletion of Staphylococcus aureus restored lung function and reduced the deleterious effects of airway dysbiosis (Liang et al., 2023). While clinical evidence remains limited, phage therapy represents an innovative strategy to correct selected components of dysbiosis and may eventually complement antibiotic therapy or reduce reliance on broad-spectrum antibiotics.

Last but not least, tracking changes in the composition and function of microbiota might allow assessment of treatment responses and identification of biological signatures associated with therapeutic efficacy. For example, shifts in microbial populations following probiotic supplementation may provide information about treatment-associated changes in the gut or airway ecosystem. However, the use of microbiome changes as validated biomarkers for guiding individual COPD treatment remains investigational and requires prospective validation.

Importantly, significant barriers must be addressed before microbiota-targeted therapies can be integrated into routine COPD management. These include effective delivery to the lower airways, patient stratification according to microbial and inflammatory endotypes, definition of clinically meaningful microbiome endpoints, and safety considerations in a chronically inflamed lung environment. Carefully designed, stratified clinical trials will be essential to determine whether microbiota modulation can provide meaningful adjunctive benefit in selected COPD subgroups.

## Knowledge gaps and future directions in microbial ecology of COPD

### Knowledge gaps

Increasing evidence demonstrates that the lower airways harbor dynamic microbial communities even in health and implicates lung dysbiosis across different COPD clinical and inflammatory phenotypes. However, major questions remain unresolved regarding microbial function, causality, temporal dynamics, host interactions, clinical relevance, and therapeutic implications (Natalini et al., 2023; Mayhew et al., 2018; Dima et al., 2019). In fact, although an increasing number of longitudinal studies are now available, many studies remain cross-sectional and are conducted in established disease, limiting conclusions as to whether dysbiosis drives pathogenesis or reflects disease stage. It remains unclear whether microbial alterations precede airflow limitation in at-risk smokers or emerge only after obstruction develops, and longitudinal data spanning the full range of COPD severity and incorporating individuals at risk of COPD remain limited (Sulaiman et al., 2023; Dima et al., 2019).

Phenotype-specific microbial signatures are also incompletely defined. Whether symptomatic patients differ microbiologically from less symptomatic individuals at similar airflow limitation, or whether frequent exacerbators exhibit stable colonization versus ecological instability, is uncertain (Sulaiman et al., 2023; Dang et al., 2022). Species-level analyses may overlook clinically relevant strain-level heterogeneity, as strains within the same species can differ in virulence, biofilm formation, antimicrobial resistance, and immunomodulatory properties. However, strain-resolved metagenomic analyses remain relatively limited in well-phenotyped COPD cohorts, potentially obscuring links between microbial variation and disease severity or treatment response (Tiew et al., 2021).

Beyond taxonomy, microbial function may be more informative. Microbial metabolites and signaling pathways can influence epithelial integrity and airway inflammation, yet integrated multi-omics studies in well-characterized COPD populations remain relatively scarce. Indeed, a multi-omic meta-analysis identified functional links between the metabolic potential of the airway microbiome and host transcriptional pathways in COPD, highlighting the importance of moving beyond taxonomic descriptions (Wang et al., 2002; Wang et al., 2020). Host-microbiota interactions further complicate interpretation: immune endotypes, inhaled corticosteroids, and chronic macrolide therapy may all shape airway ecology and microbial dynamics (Wang et al., 2021; Sulaiman et al., 2023; Keir et al., 2021; Lim et al., 2023).

Exacerbations, often , are accompanied by shifts in bacterial load and/or community composition, but whether repeated events progressively destabilize microbiota and accelerate lung function decline remains uncertain.

Methodological challenges including low microbial biomass, contamination risk, sampling variability, and heterogeneous sequencing and bioinformatic pipelines limit reproducibility and comparability across studies (Caverly et al., 2019; Fierer et al., 2025; Porcari et al., 2025). These limitations are particularly relevant to lower-airway studies, in which microbial biomass can be low and contamination from the upper airway or laboratory reagents can substantially influence results. Standardized sampling, rigorous negative controls, and harmonized analytical approaches are therefore essential for improving reproducibility.

Finally, the gut-lung axis underscores the systemic dimension of COPD; although bidirectional interactions between intestinal and pulmonary microbiota are increasingly recognized, their contribution to specific COPD phenotypes, particularly frequent exacerbators, remains insufficiently established (Wang et al., 2021; Li et al., 2021). Experimental studies suggest that COPD-associated gut dysbiosis can influence pulmonary inflammation and disease features, but the extent to which these interactions contribute to disease heterogeneity and clinical outcomes in humans remains uncertain.

The major challenge is to move from descriptive associations toward longitudinal, mechanistic, and clinically actionable models that integrate microbial composition and function with host immune and epithelial responses, environmental exposures, treatment, and COPD phenotype. This transition is essential if the double-edged nature of the lung microbiota is to be translated into reproducible biomarkers or rational microbiota-directed interventions.

### Future directions

Table 2 summarizes the current status and highlights research directions to address the gaps and potentially translate microbiota research into precision medicine strategies.

**Table 2:** Proposed list of directions in lung dysbiosis-mediated COPD pathogenesis research.

Research area	Current knowledge gap	Research priority
**Causality and temporal dynamics**	Many studies are cross-sectional and involve established COPD, making it difficult to determine whether dysbiosis precedes disease or reflects disease progression.	Conduct longitudinal studies spanning at-risk individuals, early disease, and established COPD to clarify the temporal relationship between microbial changes and disease development.
**COPD phenotypes and endotypes**	Phenotype-specific microbial signatures remain incompletely defined, including differences related to symptoms, exacerbation susceptibility, and inflammatory phenotypes.	Use well-characterized cohorts to identify reproducible microbiota signatures associated with clinically relevant COPD subgroups.
**Strain-level resolution**	Species-level profiling may obscure strain-specific differences in virulence, biofilm formation, antimicrobial resistance, and immunomodulatory properties.	Apply strain-resolved metagenomics to distinguish functionally relevant microbial variants and relate them to disease severity and treatment response.
**Microbial function and multi-omics**	Microbial functional activity is less well characterized than taxonomic composition, and integrated multi-omics studies remain limited.	Integrate metagenomics, metatranscriptomics, metabolomics, and host molecular profiling to link microbial functions with epithelial and inflammatory pathways.
**Host-microbiota interactions**	The relationships among microbial ecology, immune endotypes, and treatment exposure remain incompletely understood.	Combine microbiome analysis with epithelial and immune profiling to determine how host inflammatory states and treatments reshape microbial ecology.
**Exacerbation dynamics**	Although exacerbations are associated with changes in bacterial load and community composition, the effects of repeated exacerbations on microbial stability and lung-function decline remain uncertain.	Perform longitudinal sampling before, during, and after exacerbations to distinguish transient microbial changes from persistent ecological alterations.
**Methodological standardization**	Low microbial biomass, contamination, sampling variability, and heterogeneous sequencing and bioinformatic pipelines limit reproducibility across studies.	Establish standardized sampling procedures, negative controls, sequencing methods, and analytical pipelines for respiratory microbiome studies.
**Gut-lung axis**	The contribution of gut–lung interactions to COPD heterogeneity and specific clinical phenotypes remains uncertain.	Investigate relationships among gut dysbiosis, microbial metabolites, systemic immunity, pulmonary inflammation, and COPD outcomes using longitudinal and mechanistic approaches.
**Clinical biomarkers**	Microbial signatures associated with disease severity, exacerbations, and clinical outcomes lack sufficient external validation for routine clinical application.	Validate microbiota-derived biomarkers prospectively in multicenter cohorts and determine whether they improve prediction beyond established clinical variables.
**Therapeutic translation**	Standard COPD treatments, including inhaled corticosteroids, can alter the airway microbiota, but the clinical consequences of these changes remain unclear. Microbiota-directed interventions and microbiome-guided treatments also remain investigational.	Determine how standard COPD treatments affect the airway microbiota and clinical outcomes, and evaluate whether microbiome-guided approaches can improve treatment selection and response.

## Declaration of competing interest

The authors declare that they have no known competing financial interests or personal relationships that could have appeared to influence the work reported in this paper.
